# Pulmonary Function Tests in Hypertensive Patients Attending Zewditu Memorial Hospital, Addis Ababa, Ethiopia

**DOI:** 10.1155/2018/5492680

**Published:** 2018-11-12

**Authors:** Mekuriaw Mesfin Birhan, Yekoye Abebe

**Affiliations:** ^1^Department of Physiology, Bahir Dar University, College of Medicine and Health Sciences, Bahir Dar, Ethiopia; ^2^Department of physiology, Addis Ababa University College of Medicine and Health Sciences, Addis Ababa, Ethiopia

## Abstract

**Background:**

Hypertension imposes stresses on many organs like heart and kidney. However, studies that show the effect of hypertension on the lungs are limited.

**Objective:**

To assess pulmonary function status of hypertensive patients aged 30-64 years at Zewditu Memorial Hospital, 2017.

**Methods:**

Hospital based comparative cross-sectional study was conducted on 61 hypertensive patients (cases) and 61 nonhypertensive clients (controls) aged 30-64 years. Computerized spirometry was done in all cases and controls which were selected by systematic sampling technique. The study was conducted from January 20, 2017, to May 25, 2017. Result. The values of FVC, FEV1, and FEF25-75% were 3.52±1.02 liters, 2.97±0.89 liters, and 3.34±1.3 liters/second in hypertensive patients and 4.31±0.82 liters, 3.54±0.7 liters, and 3.94±1.09 liters/second in controls, respectively. These values were significantly lower (p<0.05) in hypertensive patients compared to controls. Restrictive pulmonary defect was dominant in hypertensive patients. FEV1% which was 85%±7% in hypertensive patients and 82%±5% in controls was significantly higher (p<0.05) in hypertensive patients compared to controls.

**Conclusion:**

Hypertensive patients exhibit lower pulmonary function values. Routine check-up of the pulmonary function status of such patients should be done to prevent undesired outcomes.

## 1. Introduction

Hypertension is an established risk factor for cardiovascular disease and an important determinant of cardiovascular risk [1].

Globally over 1 billion people are living with high blood pressure [2]. In 2008, globally, the overall prevalence of high blood pressure (including those on medication for high blood pressure) in adults aged 25 and above was around 40%. Among all WHO regions, the prevalence of raised blood pressure was the highest in the African Region (46%) and the lowest in the region of the Americas (35%) [2].

The prevalence of hypertension among Ethiopian population was estimated to be 19.6% [3]. Subgroup analyses indicated that the prevalence of hypertension is higher in the urban population (23.7 %) than rural and urban combined (14.7 %) [3].

Epidemiological data support a continuous risk of cardiovascular disease, stroke, and renal disease across levels of both systolic and diastolic blood pressure [1, 4].

Hypertension imposes stresses on many organs [2]. Complications include congestive heart failure, strokes, renal failure, and many others [5]. Spontaneous haemorrhage caused by bursting of small vessels elsewhere in the body may also occur but with less serious consequences [5].

Until complications occur, hypertension has no symptoms, because the tissues are adequately supplied with blood. Therefore, unless blood pressure measurements are made on a routine basis the condition can go undetected until a precipitous complicating event [5].

Studies that show the effect of hypertension on the lung are limited and contradicted. Some investigators show a decrease in pulmonary function parameters in hypertensive patients [6]. The pathophysiology claimed was interstitial edema of the lung secondary to left ventricular failure by high sustained blood pressure and decreased elasticity of the pulmonary parenchyma [6–8]. In contrast some other investigators concluded that hypertension has no effect on pulmonary function rather the antihypertensive medications have the above effect [9]. Despite the high prevalence of hypertension in Ethiopia, there is no research done on effect of hypertension on pulmonary function. The aim of the present study was to assess pulmonary function of hypertensive patients who attended Zewditu Memorial Hospital, Addis Ababa, Ethiopia. Establishing the effect will help health professionals to screen the complication and take appropriate measures as early as possible. The finding may also help professionals to give emphasis on pulmonary complication caused by hypertension like its complication on kidney, heart, and other organs.

## 2. Materials and Methods

Hospital based comparative cross-sectional study was conducted on 61 hypertensive patients at medical OPD or that came for follow-up at hypertension clinic of Zewditu Memorial Hospital and 61 controls (nonhypertensive) aged 30-64 years from August 20, 2016, to September 25, 2016. Systematic sampling technique was used to select both cases and controls. The sex distribution is equal in both cases and controls. The average age and anthropometric difference in cases and controls were minimal. Hypertensive patients or controls that had any cardiopulmonary diseases, neuromuscular diseases or any systemic disease, patients on *β* blockers (atenolol, metoprolol, labetalol, propranolol, carvedilol, etc.), and smokers were exclude from the study.

Respiratory system variables (FVC, FEV1, FEV1/FVC, PEFR, and FEF25-75%) were measured with digital spirometer for both controls and hypertensive patients. Participant blood pressure was measured 3 times in a sitting position using standard mercury sphygmomanometer on left upper arm at heart level. The average of the second and third BP measurements was used to determine the blood pressure of the participant.

Face-to-face interviews using semistructured questionnaire were made to collect sociodemographic data and medical history of both the cases and controls.

Data was entered and analyzed using SPSS V 20.0. One-way ANOVA, Student's* t*-test, and bivariate correlation were used for testing the strengths of the associations among variables. The association was considered significant only if p value was less than 0.05.

## 3. Result

### 3.1. Sociodemographic Data

In this study a total of 122 subjects, aged 30-64 years were recruited. Sixty-four of the subjects were females and 58 were males. Among the total subjects 61 were diagnosed as hypertensive patients (cases) and 61 were nonhypertensive relatively healthy persons (controls) (Table 1).

### 3.2. Anthropometric & Blood Pressure Measurements

The difference in mean values of age, sex, height, weight, and BMI between cases and controls was not significant (P>0.05), whereas mean systolic blood pressure (SBP) and diastolic blood pressure (DBP) were found higher in cases than controls with highly significant p value (p<0.001) (Table 2)

### 3.3. Pulmonary Function Tests

The mean difference of pulmonary function tests between cases and controls was compared using independent samples t-test. On comparison, FVC, FEV1, and FEF25-75% were significantly lower in cases than in controls (P<0.05). FEV1% was found significantly higher (p<0.05) in case group. PEFR was found lower in cases group but not significant (Table 3)

**Figure 1:**
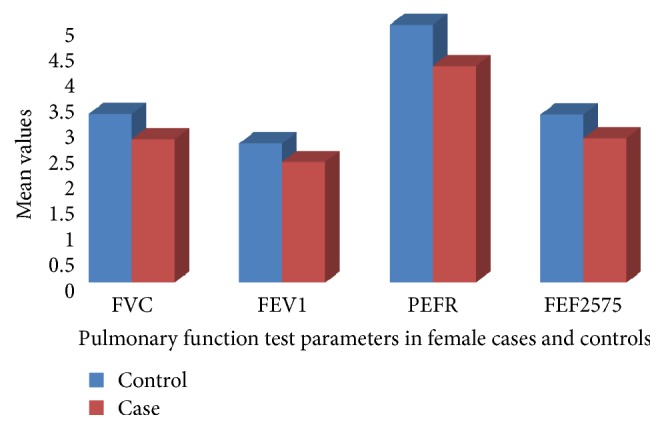
Mean values of pulmonary function tests in hypertensive (cases) and nonhypertensive (controls) females at Zewditu Memorial Hospital, Addis Ababa, Ethiopia, 2017.

**Figure 2:**
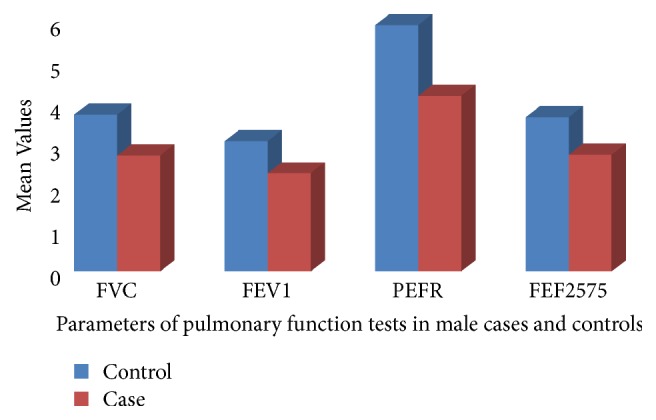
Mean values of pulmonary function tests in hypertensive females (cases) and nonhypertensive females (controls) at Zewditu Memorial Hospital, Addis Ababa, Ethiopia, 2017.

The pulmonary functions of hypertensives are compared by sex with nonhypertensive controls. In both hypertensive and nonhypertensive females FVC, FEV1, PEFR, and FEF2575 are significantly lower (*α* value < 0.05) in hypertensive females compared to nonhypertensive females. FVC and FEV1 are also significantly lower in male hypertensives than nonhypertensive male controls. PEF and FEF2575 are also lower in hypertensive males than nonhypertensive males (Figures 1 and 2)

**Figure 3:**
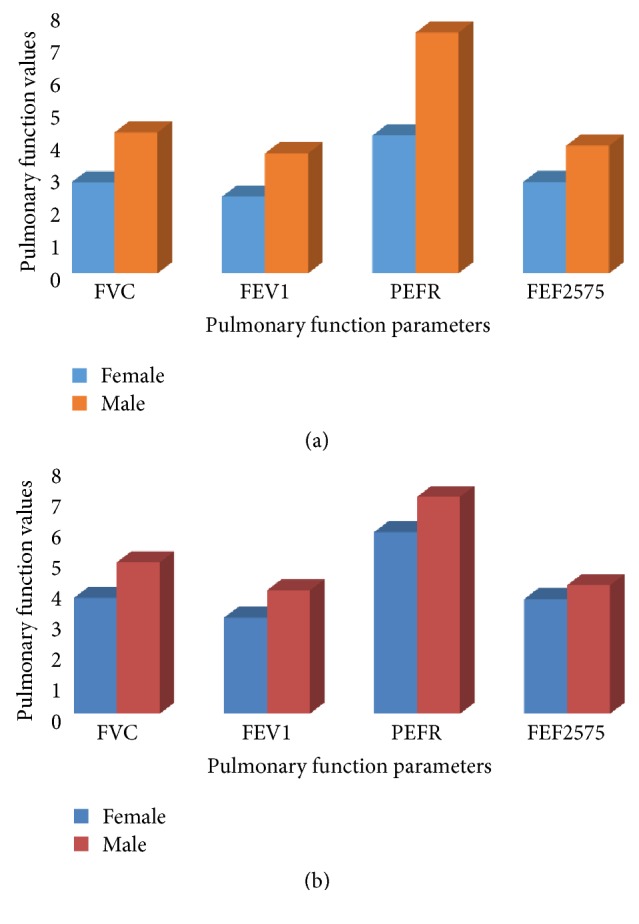
Mean values of pulmonary function tests in hypertensive females and hypertensive men at Zewditu Memorial Hospital, Addis Ababa, Ethiopia, 2017.

The pulmonary function of male hypertensive patients is significantly higher (p value <0.05) than female hypertensive patients (p value <0.05) (Figure 3(a)). The same is true for controls; the pulmonary functions of nonhypertensive males is significantly higher than nonhypertensive females (Figure 3(b)); so in both cases and controls females have lower values of pulmonary functions compared to males.

The observed PFT values were compared with the predicted values considering the patient's age, sex, weight, and height. In hypertensive patients (cases), the observed values of FVC, FEV1, and PEFR were significantly lower (p<0.05) than the predicted values. The observed values of FEV1% and FEF25-75% were higher than the predicted values but only the former is statistically significant (p<0.05). In control groups the observed values of FVC, FEV1, FEV1%, and FEF25-75% were higher than the predicted values but all these were not statistically significant (Table 4).

To see the association between mean blood pressure and different pulmonary function indices, bivariate correlation was made. FVC and FEV1 had weak negative correlation but FEV1%, PEFR, and FEF25-75% had no significant correlation (Table 5).

**Figure 4:**
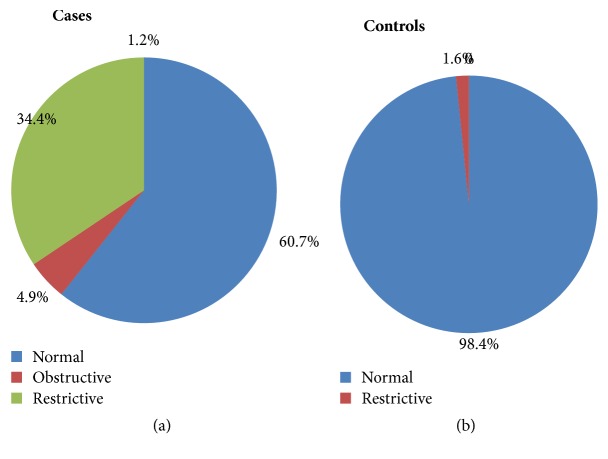
Pulmonary function status in hypertensive patients (a) and nonhypertensive persons (b) at Zewditu Memorial Hospital, Addis Ababa, Ethiopia, 2017.

Among 61 hypertensive patients, 24(39.3%) had pulmonary function disorders (34.4% restrictive and 4.9% obstructive). From 61 controls, 1(1.6%) person had restrictive disorder (Figure 4).

Among 122 study subjects, 22 (18%) had percent predicted FVC value below 80% and 14 (11.5%) had percent predicted FEV1 value below 80%. All the subjects that had percent predicted FVC and FEV1 values below 80% were hypertensive patients except one control that had percent predicted FVC value below 80% (Table 6).

## 4. Discussion

In the present study, the difference in mean values of age, sex, height, weight, and BMI between hypertensive patients and controls was not significant (Table 2); therefore, it is possible to compare the pulmonary function parameters in the two groups.

In this study, FVC, FEV1, and FEF25-75% were found significantly lower (p<0.05) in hypertensive patients compared to controls in both sexes (Table 2, Figures 1 and 2). This was in line with other study that showed the respiratory parameters, i.e., FVC, FEV1, FEV1/FVC%, PEFR, and FEF25-75, were significantly lower in HT patients when compared with control population [10].

Similar studies also concluded that hypertensive subjects had significantly lesser values of FVC, FEV1, FEV1/ FVC, and PEFR as compared to normotensive subjects [6, 11]. Another study showed only medication (not hypertension) decreased FEV1 and FVC significantly [9].

In this study, FEV1% was found significantly higher (p<0.05) in hypertensive patients than controls (Table 4). This may be due to the fact that FVC was more affected than FEV1 in cases group. Higher FEV1% in hypertensive patients also indicate restrictive pattern of pulmonary function.

In this study FVC, FEV1, PEFR, and FEF25-75 were significantly lower in both hypertensive and nonhypertensive females compared to hypertensive and nonhypertensive males (Figure 3). This may be due to the fact that females usually have lesser values of these variables compared to males due to physical difference [12]. The finding is also similar to the finding by other study [11].

In hypertensive patients (cases), the observed values of FVC, FEV1, and PEFR were not only lower than the controls but also significantly lower (p<0.05) than the predicted values (Table 4). This finding was similar to the finding by other study [13].

In this study, no significant association found between pulmonary function and severity of hypertension (Table 5). This finding is similar to the finding by other studies [11]. The lack of association in the present study may be due to the fact that many hypertensive patients are on antihypertensive drugs that might have inhibited the effect of hypertension.

In this study, restrictive lung disease was highly prevalent (34.4%) in hypertensive patients compared to the controls (1.6%) (Figure 4). There was also 4.9% obstructive lung disorder in hypertensive patients but not in control. In line with this study, other studies also showed restrictive pattern was dominant [11, 13] but another study showed 74% hypertensive patients were having obstructive pattern, while 13% of the patients were having restrictive pattern of their pulmonary function test parameters [10]. According to that study the dominant pulmonary defect in hypertensive patients was obstructive [10]. The reason for the difference with this study may be due to the inclusion of hypertensive patients taking beta blockers which cause bronchoconstriction in that study. In the current study, hypertensive patients on beta blockers were excluded from the study.

In the present study mean blood pressure showed a weak correlation with some pulmonary function parameters (Table 5). FVC and FEV1 had negative weak correlation (r= -0.243 and -0.221, respectively) but FEV1%, PEFR, and FEF25-75% had very weak or no correlation at all (Table 6). The weak or lack of correlation may be due to the fact that most of the hypertensive patients were taking antihypertensive medication and HT is controlled in many of them. Other studies showed that mean blood pressure had no significant correlation with FVC and FEV1 [10]. The possible reason claimed for this was the small sample size investigated (N=30 for cases and 30 for controls). In this study the total study subjects were 122 and equal sizes for cases and controls.

Generally in this study the null hypothesis is rejected and the alternative hypothesis is accepted. This means there is significant association between hypertension and pulmonary function.

## 5. Conclusion

Hypertensive patients exhibit lower pulmonary function values. FVC, FEV1%, FEF25-75%, and PEFR were significantly lower in hypertensive patients compared to controls. The pulmonary function parameters of hypertensive patients were also significantly lower than the percentage predicted values. In this study restrictive lung disease was the dominant pulmonary defect in hypertensive patients. PFTs can be used as an integral part of screening methods for hypertensive patients and thereby improve their quality of living by retarding progression to complications. Hypertension can also be considered as a risk factor for impaired pulmonary function.

## Figures and Tables

**Table 1:** Sociodemographic characteristics of study participants at Zewditu Memorial Hospital, Addis Ababa, Ethiopia, 2017.

Variable	Hypertensive			
	patients	Controls	Total	
	( N )	(N)	(N)	%
Sex				
Female	32	32	64	52.5
Male	29	29	58	47.5

Age(years)				
30-34	1	3	4	3
35-39	2	2	4	3
40-44	2	9	11	9
45-49	11	9	20	16
50-54	21	21	41	33.6
55-59	15	12	27	22
60-64	9	5	14	11.4

Marital status				
Single	7	4	11	9
Married	44	50	94	77
Divorced	8	4	12	9.9
Widowed	2	3	5	4.1

Educational status				
Illiterate	1	1	2	1.6
Read &write(informal)	4	5	9	7.4
Primary school (1-4)	4	0	4	3.3
Primary school (5-8)	21	15	36	29.5
Secondary school (9-12)	20	23	43	35.2
Certificate	2	4	6	4.9
Diploma & above	9	13	22	18

**Table 2:** Physical characteristics of hypertensive patients and controls at Zewditu Memorial Hospital, Addis Ababa, Ethiopia, 2017.

Parameter	Hypertensive patient N=61 Mean ± SD	Control N=61 Mean ± SD	P-value
Age (yrs)	52 ± 6.66	50 ± 7.5	.131

Weight (kg)	69 ± 11	70 ± 8.36	.345

Height (cm)	162 ± 7.28	164 ± 7.44	.077

BMI (kg/m2)	25.88 ± 2.94	25.81 ± 2.41	.885

SBP (mmHg)	143 ± 20	115 ± 6	< 0.001*∗∗*

DBP (mmHg)	89 ± 11.3	74 ± 9	< 0.001*∗∗*

Mean BP (mmHg)	107±13.58	88±7.23	< 0.001*∗∗*

*∗∗* P value <0.01 is considered highly significant. BMI: body mass index, SD: standard deviation, SBP-systolic blood pressure, and DBP: diastolic blood pressure.

**Table 3:** Comparison of PFTs in hypertensive patients and controls at Zewditu Memorial Hospital, Addis Ababa, Ethiopia, 2017.

Parameter	Hypertensive patients Mean ± SD	Controls Mean ± SD	P-value
FVC (liters)	3.52 ± 1.02	4.31 ± 0.82	< 0.001*∗∗*

FEV1 (liters)	2.97 ± 0.89	3.54 ±0.7	< 0.001*∗∗*

FEV1/FVC %	85 ± 7	82± 5	.034*∗*

PEFR (liter/sec)	5.70 ± 2.44	6.44 ± 2.07	.076

FEF2575 (liter/sec)	3.34 ± 1.30	3.94 ± 1.09	.007*∗∗*

*∗*P value< 0.05 is considered significant and *∗∗*P value < 0.01 is considered highly significant; SD: standard deviation.

**Table 4:** Observed and predicted PFTs in hypertensive patients and controls at Zewditu Memorial Hospital, Addis Ababa, Ethiopia, 2017.

PFT Parameter (mean values)	Observed	Predicted	P-value
FVC(L)			
Case	3.52 ± 1.02	3.86 ± 0.67	< 0.001*∗∗*
Control	4.31 ± 0.82	4.21 ± 0.71	0.373
FEV1 (L)			
Case	2.97 ± 0.89	3.14 ± 0.61	0.046*∗*
Control	3.54 ± 0.7	3.37 ± 0.61	0.124
FEV1%			
Case	85 ± 7	78.18 ± 3.07	< 0.001*∗∗*
Control	82 ± 5	79.82 ± 4.08	0.152
PEFR (L/S)			
Case	5.70 ± 2.44	7.28 ± 1.37	< 0.001*∗∗*
Control	6.44 ± 2.07	7.87 ± 1.67	< 0.001*∗∗*
FEF25-75% (L/S)			
Case	3.34 ± 1.30	2.9 ± 0.57	0.068
Control	3.94 ± 1.09	3.47 ± 1.06	0.118

*∗*p value<0.05 is significant; *∗∗*p value<0.01 is highly significant.

**Table 5:** Bivariate correlation result between pulmonary function parameters and mean blood pressure in cases and controls at Zewditu Memorial Hospital, Addis Ababa, Ethiopia, 2017.

Parameter	Correlation coefficient (r)	p-value
Mean BP with		
FVC	-0.243	0.007*∗∗*
FEV1	-0.221	0.014*∗*
FEV1%	0.042	0.644
PEFR	-0.119	0.193
FEF25-75%	-0.145	0.110

*∗∗*p value <0.01 is highly significant; *∗* p value <0.05 is significant. BP: blood pressure.

**Table 6:** Number of cases and controls on different ranges of percentage of predicted values of FVC, FEV1, and FEF25-75% at Zewditu Memorial Hospital, Addis Ababa, Ethiopia, 2017.

PFT parameter & its range	Hypertensive patients		Control	
	N	Percentage	N	Percentage
%predicted FVC				
≥80%	40	32.8	60	49.2
<80%	21	17.2	1	0.8
%predictedFEV1				
≥80%	47	38.5	61	50
<80%	14	11.5	0	0
%predictedFEF25-75%				
≥60%	60	49.2	61	50
<60%	1	0.8	0	0

## Data Availability

The data used to support the findings of this study are available from the corresponding author upon request.
